# Introducing and testing the maternal vulnerability segmentation tool (MVST) for essential health services use: demonstrating its use in Oromia, Ethiopia

**DOI:** 10.1136/bmjgh-2024-018811

**Published:** 2026-01-07

**Authors:** Yihunie Lakew, Habtamu Tamene, Bee-Ah Kang, Nandita Kapadia-Kundu, Simon Heliso Kuka, Rajiv Rimal

**Affiliations:** 1 Center for Communication Programs, Johns Hopkins University, Addis Ababa, Ethiopia; 2 Department of Health, Behavior and Society, Johns Hopkins University, Baltimore, MD, USA

**Keywords:** Maternal health, Screening

## Abstract

Ethiopia has made substantial progress in improving maternal health outcomes; however, significant disparities remain among populations whose individual, interpersonal, community and cultural factors place them at greater risk, as compared with those with better access to resources and healthcare facilities. Addressing these disparities requires initiating advanced and targeted segmentation tools to effectively identify and reach vulnerable groups.

This study developed and validated a maternal vulnerability segmentation tool designed to assist community health workers to identify pregnant women who are least likely to access essential maternal care services. Key predictors of maternal health service utilisation were identified and incorporated into an initial 20-item questionnaire. Through a process of refinement and validation, these were condensed into six items for rural and four for urban–rural settings. Findings indicated that women with higher vulnerability scores were significantly less likely to attend antenatal care and more likely to deliver at home (p<0.01, for each). Based on these results, a maternal vulnerability segmentation tool was tested and implemented by community health workers to identify and support underserved pregnant women in Ethiopia.

**WHAT IS ALREADY KNOWN ON THIS TOPIC**

- Nearly half of Ethiopian pregnant women are not using essential maternal services such as antenatal care and health facility delivery, placing their newborn and own health at risk.
- Pregnant women are less likely to use those services because of individual, social, economic and environmental factors.

**WHAT THIS STUDY ADDS**

- This study provides new maternal vulnerability segmentation tools to identify women living with vulnerabilities for targeted interventions.
- It explains the extent to which intersecting vulnerability scores affect essential maternal service utilisation.

**HOW THIS STUDY MIGHT AFFECT RESEARCH, PRACTICE OR POLICY**

- This study enables community health workers to classify pregnant women according to their degree of vulnerability, thus paving the way for developing well-targeted interventions.
- It also calls for further research aimed at generating more validated vulnerability segmentation tools that effectively and efficiently address maternal vulnerabilities across different contexts in different Ethiopian regions and other low-income and middle-income countries.

## Introduction

 Ethiopia has shown significant progress in many maternal and child health indicators over the past few decades. For example, in 2000, the maternal mortality ratio (MMR) was 871 deaths per 100 000 live births and declined to 412 in 2016.^1^ This achievement is largely attributed to the government’s health initiatives^2^ that improved the coverage of maternal and child health services, such as antenatal care (ANC) and health facility delivery.^3^ Indeed, timely access to maternal child health services has been shown to reduce maternal deaths by between 16% and 33%^4^ and neonatal mortality by 29%.^5^

However, women’s use of health facilities for childbirth greatly varies by region and sociodemographic characteristics. With the national average being approximately 48%, the percentage of institutional delivery (health facility delivery) ranges from 23.3% in Somali Region to 94.8% in Addis Ababa, the capital city.^6^ The percentage of health facility delivery in Oromia region (the site of this study) is 41%, lower than the national average.^6^ Also, 79% of women in the lowest wealth quintile delivered a baby at home; the corresponding figure for women in the highest wealth quintile was 14%. This implies appropriate action is required to address such disproportionate uptake of and access to maternal health services.

Reducing disparities in access to essential services requires strategies tailored to the specific geographical, social and health contexts. One such strategy is to develop a vulnerability segmentation tool, which identifies factors that put women at risk, and then tailor interventions accordingly. Vulnerability segmentation is becoming an important concept in such efforts.^7 8^ Despite its importance, less attention has been paid to how vulnerability is defined, conceptualised and operationalised in the realm of maternal health service coverage.^8^ There is also a lack of consensus on what constitutes vulnerability and the underlying multidimensional factors that comprise it.^8 9^

A substantial body of literature exists on vulnerabilities derived from geographical, economic, environmental and social contexts. These vulnerabilities, often manifesting as disparities, are typically viewed through two lenses: the behavioural perspective and the structuralist perspective.^10^ Other perspectives also exist, including the distinction between external and internal dimensions of vulnerability, where the external refers to exposure to risk and the internal refers to individuals’ capacities to cope with stressors and outcomes.^11 12^ Few scoping reviews also define vulnerability in the context of maternal and child health with factors broadly categorised into three themes: socioeconomic, biological and environmental.^7 13^

Furthermore, certain vulnerability indexes are available, including those measuring economic, socio-environmental, drought and heat-related vulnerabilities.^14^,^16^ Though of great utility, these indexes are primarily intended for national and subnational use, and their applicability to community, household or individual levels is limited. For example, in the USA, there is a maternal vulnerability index (MVI) available at county and state levels.^17^ It was developed by Surgo Ventures and has 43 indicators across six themes: (1) reproductive healthcare, (2) physical health, (3) mental health and substance abuse, (4) general healthcare, (5) socioeconomic determinants and (6) physical environment. Although the MVI is useful for identifying geographies with high or low maternal risk, it cannot identify households and individuals using those multidimensional vulnerability drivers.^17^ MVI’s application includes research to show the index’s association with adverse health outcomes, which generates evidence of the association between vulnerability and poor maternal outcomes. Other maternal vulnerability scales also have shown limitations to effectively segment degrees of vulnerability at household and individual level.^15^,^18^

Segmenting pregnant women by degree of vulnerability enhances the scope of providing equitable maternal health services in a cost-effective manner. Also, as found in the literature, there is a lack of practical guidance on how to identify specific households and individuals with maternal vulnerability at the community level, a simple segmentation tool is required for frontline health workers to directly and timely address their specific needs. The rationale of developing an evidence-based ‘maternal vulnerability segmentation tool’ (MVST), then, is to identify pregnant women who are at higher risk of failing to deliver at health facilities and use an intervention tailored to their circumstances. Therefore, the objectives of the study are twofold: (1) to develop an MVST for essential maternal health services use, and (2) to test if relationships exist between degree of vulnerability and essential maternal health services use.

## Methods

Data for this study, collected at baseline before the intervention, come from a community-based quasi-experiment run in Oromia, Ethiopia in early 2024. The study was designed as a preintervention and postintervention among a panel of pregnant women. Within this region, two woredas (administrative units below one and then region) were selected as the intervention arm and two others as the control arm. The intervention arm received behavioural solutions, whereas the control arm did not (it was conceptualised as usual care). Data collection modalities and frequencies were the same in both arms: we collected data at baseline, before the start of the intervention and then again at end-line, after the intervention. Details are indicated elsewhere.^19^

Selection of the woredas for inclusion in our study was based on the fact that they are located in Oromia, the most populous region that shares borders with all regions in Ethiopia except Tigray region, and also the most diverse, in terms of ethnicity and religion. Within Oromia, we selected the woredas to maximise the balance in sociodemographic characteristics between the intervention and control arms. Lastly, we also considered issues of safety and security; at the time of data collection, many parts of Ethiopia were undergoing political and social upheaval, making it difficult for our field staff to access many other parts of the country without sacrificing their own safety.

At baseline, we reached a total of 1614 households to establish a sampling frame and to identify pregnant women through house-to-house visits using a simple pregnancy screening tool and finally resulted in 1189 pregnant women with greater than or equal to 5 months of pregnancy. All pregnant women (5+ months of pregnancy) were listed as eligible for vulnerability segmentation and we conducted a face-to-face interview using a 20-item questionnaire, an automated digital data collection tool. After segmenting vulnerable pregnant women, we randomly sampled three women per enumeration area to achieve our target sample size for the panel study (pregnant women living with vulnerabilities). If an enumeration area had three or fewer eligible women, they were all contacted for recruitment. If there were more vulnerable pregnant women than the minimum required number of pregnant women, we randomly selected the required number. Then, 470 samples of pregnant women (5+ months of pregnancy and living with vulnerabilities) were enrolled for the baseline assessment. This study analysed two data sets: data prepared for sampling frame (n=1189) and data collected for baseline (n=470), a subset of the sampling frame. We used data from the sampling frame for the exploratory and confirmatory factor analysis, and baseline data for validation analysis to show association between vulnerability scores (the latent variable) and outcome measures (ANC and health facility delivery).

### Item development procedures

Adapting Project Pathways’ population-based vulnerability segmentation approach,^16 20^ we conducted an extensive desk review and secondary data analysis using the 2019 Ethiopia Demographic Health Survey. The data analysis was undertaken to understand context-specific drivers of maternal vulnerability affecting uptakes and utilisation of health services such as ANC and health facility delivery for pregnant women. This secondary analysis identified the predictors of maternal health service utilisation. These included women’s illiteracy, high parity, decision-making power, exposure to media and lack of household assets. A multivariate cluster analysis (K-medoids) was performed to assess these predictors’ ability to segment households and to classify individual pregnant women as low, moderate and highly vulnerable. The K-medoids clustering algorithm measures distance in multiple dimensions, representing a number of different categories or variables.^21^

We then developed a 20-item structured questionnaire from the predictors identified during literature review and secondary data analysis to serve as an MVST. The MVST encompasses three dimensions: economic drivers (eight items), social drivers (seven items) and, information and service access (five items). The questionnaire included individual, family, service-related and environmental determinants. Individual-level factors included women’s literacy, work status and ownership of a mobile phone. Family-level factors included household income, ownership of assets such as livestock and land, ownership of radio, number of children, husband’s educational status, household experience of food shortage, household decision-making autonomy and husband’s engagement in household chores. Service-related factors included household visits by health extension workers (HEWs), distance from health facility and residence in urban or rural areas. The tool was pretested to confirm its feasibility. The 20-item questionnaire applied to our context is shown in table 1 with a shorter version.

**Table 1:** Distribution of participant responses by item (n=1189)

Item code	Questions/items	Response options	Percent
vsq1	Place of residence	Urban	16.9
		Rural	83.1
vsq2	Head of the household	My husband (men)	98.3
		Myself (women)	1.7
vsq3	Marital status	Married	98.3
		Single/dissolved	1.7
vsq4	Women’s occupation	Working for income	24.6
		Not working for income	75.4
vsq5	Husband’s occupation	Working for income	92.8
		Not working for income	7.2
vsq6	Women’s education	Read and write	24.6
		Not read and write	75.4
vsq7	Husband’s education	Read and write	62.8
		Not read and write	37.2
vsq8	Number of children	Less than 4	75.5
		Greater than or equal to 4	24.5
vsq9	Size of land for farming	More than 2 *Timad*	35.3
		Two or less *Timad*	64.7
vsq10	House made from	Corrugated iron	45.3
		Not corrugated iron	54.7
vsq11	Livestock ownership	Have livestock	37.8
		Have no livestock	62.2
vsq12	Household mobile phone ownership	Having mobile at home	81.3
		Have no mobile at home	18.7
vsq13	Women’s mobile phone ownership	Women have mobile	23.0
		Women have no mobile	77.0
vsq14	Household radio ownership	Have radio	13.9
		Have no radio	86.1
vsq15	Money to buy medical drugs	Have money	47.6
		Have no money	52.4
vsq16	Household face shortage of food in the past 3 months	No shortage of food	64.5
		Experienced shortage	35.5
vsq17	Home visits by HEWs in the past 6 months	Visited by HEWs	12.0
		Not visited by HEWs	88.0
vsq18	Distance to the health facility	Near to health facility <30 m	49.6
		Far from health facility ≥30 m	50.4
vsq19	Decision-making to purchase items	Decide myself	52.9
		Decide by husband	47.1
vsq20	Husband’s support of household chores	Have husband support	38.0
		No husband support	62.0

All answer choices signifying greater wealth and facilitating favours were coded as 0 and factors contributing to vulnerability were coded as 1.

HEWs, health extension workers.

### Item reduction process

We applied exploratory factor analysis (EFA) to create reliable latent variables (factors). Orthogonal varimax type rotation was used to interpret factors that depicted correlation between observed variables and the factor.^22^ In the EFA, the eigenvalues and scree plot were considered as criteria to select the optimal number of factors. Then variables or items with rotated factor loading (>0.3) were retained. Correlation coefficients between variables were used to evaluate the magnitude and direction of relationships. Even though both factor analysis and principal component analysis are item reduction techniques, we found the principal component method inappropriate because the unique values were not zero for each item.^23^

Considering our binary nature of data along with large sample and items, root mean square error of approximation (RMSEA) values of 0.01–0.08,^24 25^ comparative fit index (CFI) greater than 0.90^26^ and Tucker-Lewis Index (TLI) value >0.9^26^ criterion were adopted for the confirmatory factor analysis to assess acceptable structural equation model (SEM) and goodness-of-fit. For sampling adequacy of the EFA, we used the Kaiser-Meyer-Olkin (KMO) test (KMO>0.50) and Bartlett’s test of sphericity with p value *<*0.05.^27^ The χ² values were not considered as a reliable measure of goodness of fit with the large sample size of 1189, since χ² could be inflated by large sample size.^27^

### Internal reliability test and threshold

Different methods and cut-off points for estimation of internal reliability coefficient exist in literature. A generally accepted rule is that Cronbach alpha coefficient of 0.60–0.95 is designated as an acceptable level of reliability with average corrected item-total correlation greater than 0.3.^28^,^31^ Moreover, the Kuder Richardson (known as KR-20 formula), an equivalent of Cronbach alpha statistic for binary items, suggested an alternative method to assess internal consistency of the items with a level of >0.5, good reliability coefficient.^32^

Evidence suggests that a scale in the preliminary stages of development is generally not thought to require the reliability of one used to discriminate between groups or of one being used to make decisions about individual segmentation.^33^ A study suggested that researchers must be mindful of context, as acceptable values of reliability depend on the purpose for which the test is being used.^25^ Considering these suggestions along with our binary data, both Cronbach alpha level of 0.60 or greater and the KR-20 coefficient having 0.5 or greater criteria with average corrected item-total correlation were used to check internal reliability. Items with internal reliability were explored for rural, urban and both urban–rural settings.

Finally, items demonstrating acceptable internal reliability in rural, urban and urban–rural settings were used to test associations with ANC attendance and institutional delivery through mediation regression analysis. This analysis examined both the direct and indirect effects of vulnerability scores on health facility delivery, mediated through ANC visits. In the mediation regression analysis, we controlled for other potential confounders (online supplemental figure).

Prediction analysis was not conducted for the urban setting due to the limited number of cases. Furthermore, an odds-of-trend analysis was performed to test the predictive capacity of vulnerability scores regarding ANC and health facility delivery. All statistical analyses were conducted using Stata V.18 software.

**Figure 1:**
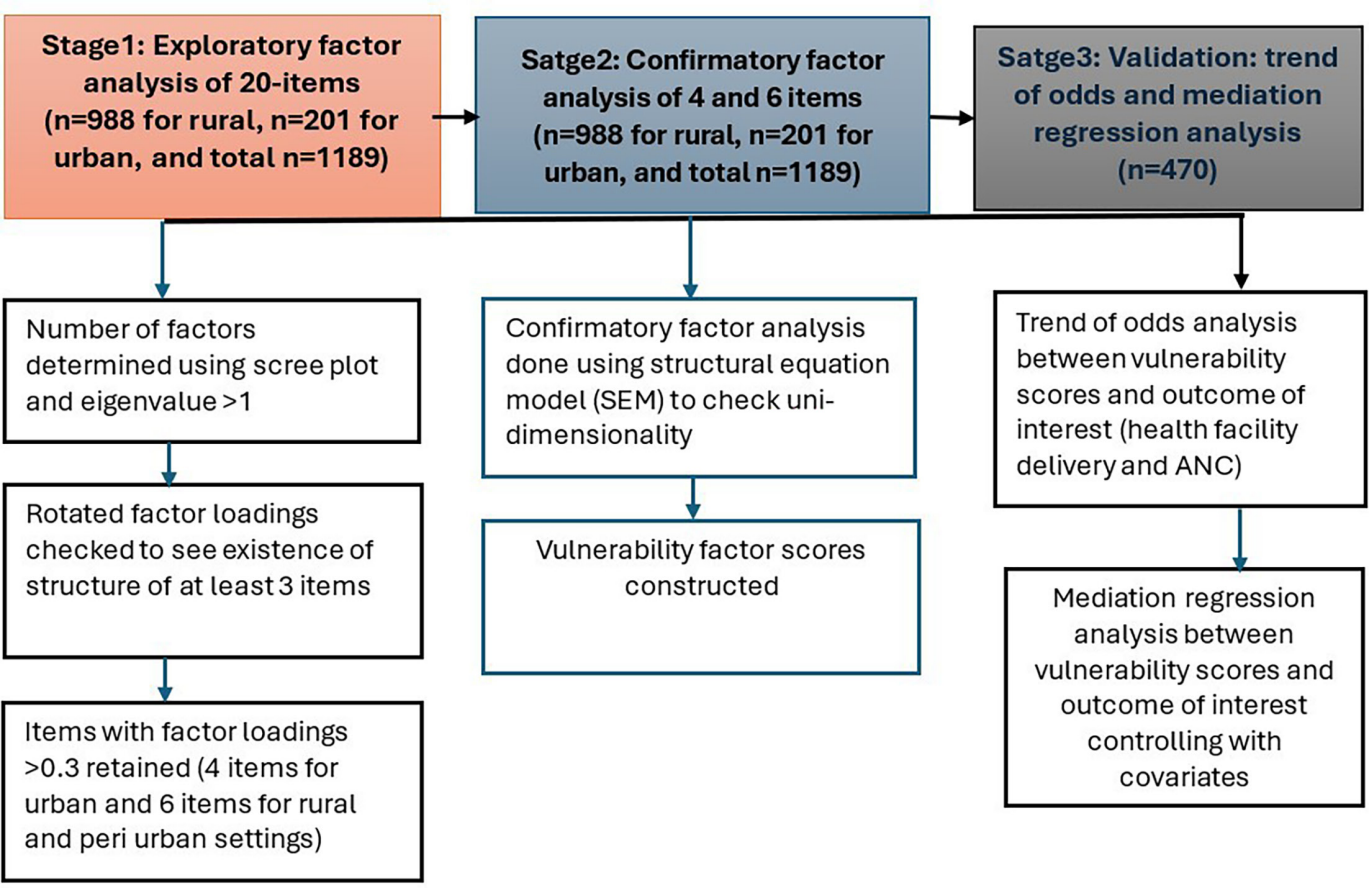
Procedures of testing the MVST in three stages of analysis including item reduction criteria and strategies, Ethiopia 2025.

Our analysis goal was to create and test the MVST to determine the extent to which it can serve as a practical and useful audience segmentation device. A flow chart summarising our analysis stages and procedures is indicated (figure 1).

### Patient and public involvement

This project involved beneficiaries (they are not technically ‘patients’) right from the beginning, when we conducted human centred design work, which informed the study design, approach, methods and the intervention itself. Different questions that comprised the MVST were also tested with participants before the decision to include them was finalised.

## Results

### Characteristics of respondents

A total of 1189 households having pregnant women were assessed using our MVST. Most of the interviewed pregnant women (83.1%) lived in rural areas. Almost all households (98.3%) were male-headed. The majority of the interviewed women (75.4%) could not read or write and did not work for income. A majority (77.0%) of the women had no functional mobile phone.

More than half of the households (62.2%) had no livestock. Nearly a third of the households (35.5%) experienced food shortages over the past 3 months. Most households (88.0%) have not received a visit from HEWs in the past 6 months. Almost half of the women (50.4%) lived more than 30 min away from a health facility. Additionally, in nearly half of the households (47.1%), only the husband decided on the purchase of major household items. About one in five households (18.7%) had a mobile phone owned by any household member. Approximately a third of husbands (37.2%) could not read or write. A majority of women (62.0%) reported that their husbands did not assist them with household chores (table 1).

The outputs from factor analysis using scree plot indicated that only one factor had a high eigenvalue of one. We retained four items for urban, four items for urban–rural and six items for rural setting that could have theoretical and practical relevance and good-to-strong reliability for the vulnerability segmentation tool. The four items for the urban–rural had factor loadings above 0.3. These items included women’s rural residence, houses not made of corrugated iron, women’s lack of mobile phone ownership and far distance from the health facility. These items provided an average interitem correlation of 0.26 and internal reliability coefficient (Cronbach’s alpha) of 0.60. Further, we analysed for the rural setting and revealed six items with a factor loading above 0.3. These include women and husbands’ lack of education, houses not made of corrugated iron, women’s lack of mobile phone ownership, households’ lack of mobile phone ownership and far distance from the health facility. These items provided an average interitem correlation of 0.20, while the internal reliability coefficient (alpha) reached 0.60. Similarly, for the urban setting, four items were identified that include women’s education, husband’s education, mobile ownership in the household and women’s mobile ownership that provided an average interitem correlation of 0.22 and internal reliability coefficient 0.52 (table 2).

**Table 2:** Reduced survey items and internal reliability coefficients from factor analysis across rural, urban and urban–rural settings (n=1189)

Retained items after rotation	Item loading	Internal reliability statistics				
	Factor loadings >0.3	Interitem correlation	Item-rest correlation	Average interitem correlation	Cronbach alpha coefficient	KR-20 coefficient
Rural setting						
Women’s education	0.47	0.60	0.37	0.20	0.55	
Husband’s education	0.42	0.57	0.33	0.21	0.56	
House made from	0.50	0.61	0.38	0.19	0.54	
Women mobile ownership	0.41	0.56	0.31	0.21	0.57	
Mobile ownership in home	0.52	0.61	0.38	0.19	0.54	
Distance to health facility	0.33	0.52	0.27	0.22	0.59	
Test scale				**0.20**	**0.61**	**0.60**
Urban setting*						
Women’s education	0.45	0.62	0.29	0.23	0.48	
Husband’s education	0.43	0.66	0.34	0.20	0.43	
Mobile ownership in home	0.35	0.58	0.22	0.27	0.53	
Women mobile ownership	0.60	0.71	0.42	0.16	0.36	
Test scale				**0.22**	**0.52**	**0.53**
Urban–rural setting						
Place of residence	0.60	0.73	0.46	0.21	0.44	
House made from	0.65	0.72	0.45	0.22	0.45	
Women mobile ownership	0.37	0.62	0.30	0.31	0.57	
Distance to health facility	0.35	0.61	0.28	0.32	0.58	
Test scale				**0.26**	**0.60**	**0.60**

* We refer to urban means a district town resident and because of small urban cases we did not do prediction analysis.

KR-20, Kuder-Richardson.

**Figure 2:**
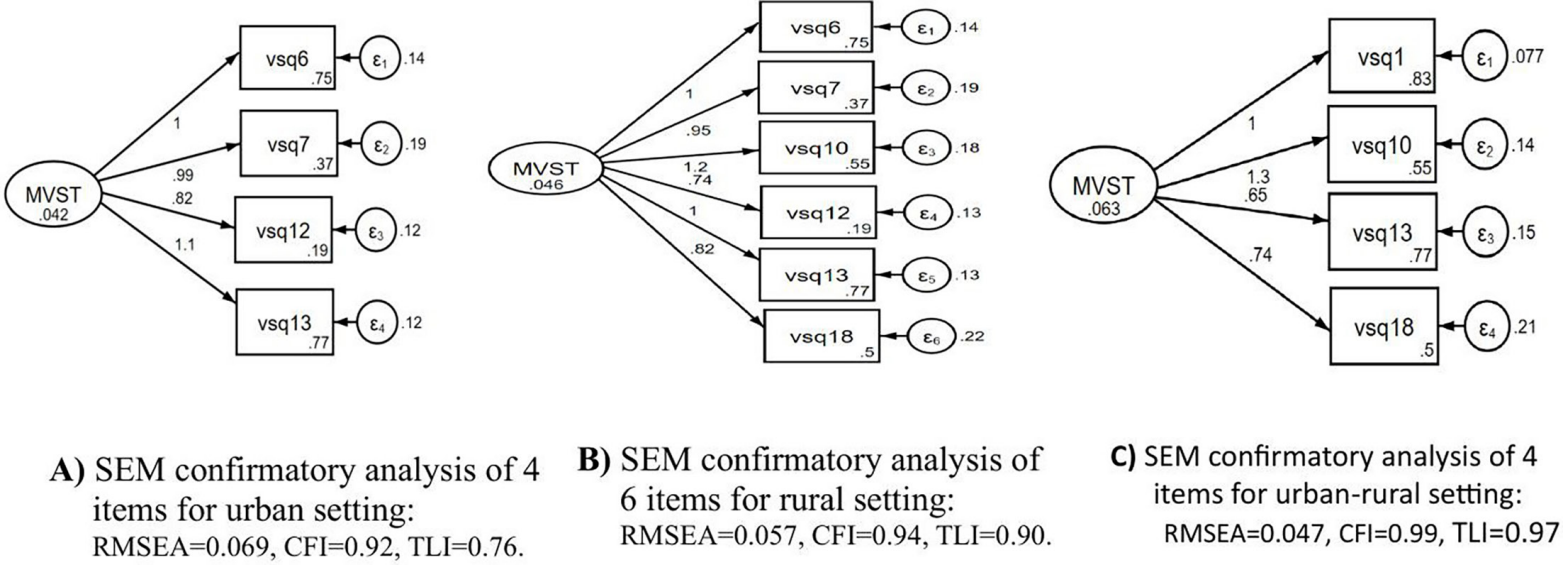
Items retained following confirmatory factor analysis using SEM (n=1189). Rotated factor structures for urban (4 items, figure 2 A), rural (6 items, figure 2 B) and urban–rural (4 items, figure 2 C) settings. CFI, comparative fit index; MVST, maternal vulnerability segmentation tool; RMSEA, root mean square error of approximation; SEM, structural equation modelling; TLI, Tucker-Lewis Index.

In our analysis of a SEM with six items for rural, four items for urban and four items for urban–rural settings, the goodness-of-fit statistics were within an acceptable range: root mean square error approximation (RMSEA)=0.047, CFI=0.99 and TLI=0.97 for all items in urban–rural settings and were significantly correlated at p<0.001. Similarly, the goodness-of-fit statistics for the six items for rural setting were found within the acceptable range: RMSEA=0.057, CFI=0.94 and TLI=0.90. Thus, the confirmatory test findings suggest that the four and six items provided an acceptable level to create a latent variable for both rural and urban–rural settings, which suggests that items showed unidimensional constructs towards the latent variables of the maternal vulnerability screening tool (figure 2).

**Figure 3:**
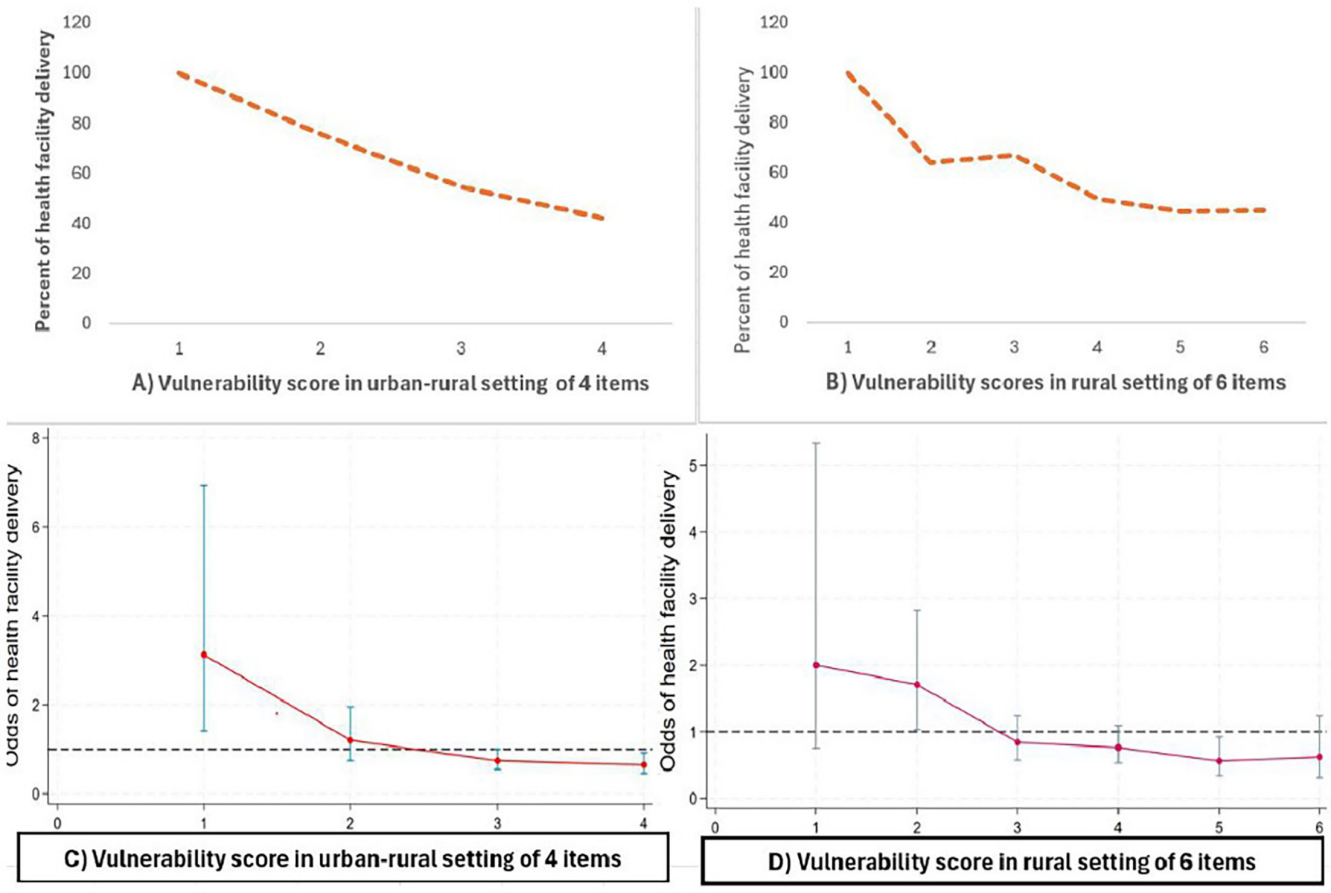
Odds of the association between health facility delivery and vulnerability scores. Trends among urban–rural women are indicated in A and C, and trends among rural women are indicated in B and D.

**Figure 4:**
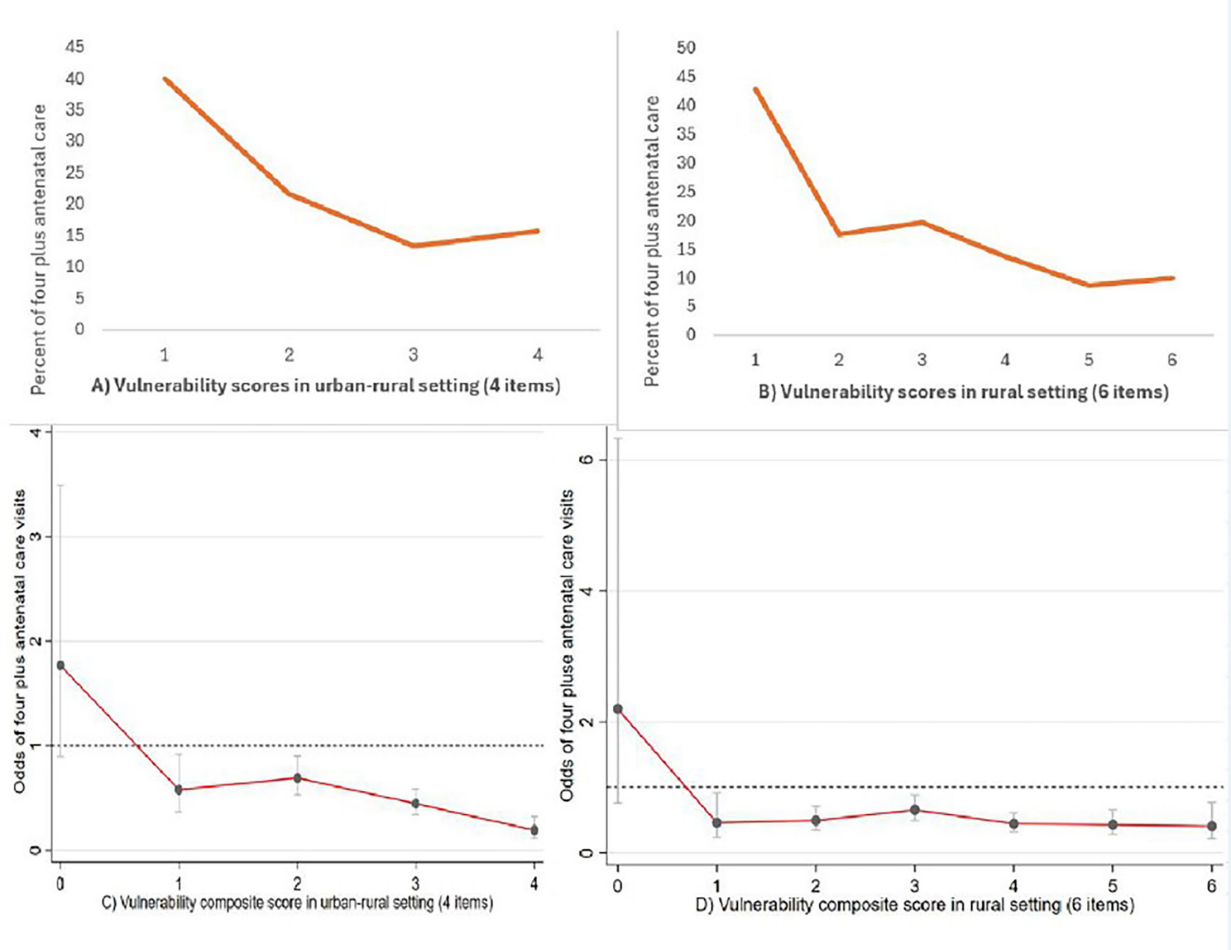
Odds of the association between antenatal care visit and vulnerability scores. Trends among urban–rural women are indicated in A and C, and trends among rural women are indicated in B and D.

As indicated in figure 3A-B, women in low vulnerability groups had a higher percentage of health facility delivery compared with those in higher vulnerability groups. As shown in figure 3C-D, we observed a declining trend in health facility delivery with an increase in a composite vulnerability score of four and six item points starting from an intersecting score of 3 points (p<0.001). These findings indicate that the four and six items can segment pregnant women in urban–rural and rural settings, respectively, for women who are at higher risk of institutional delivery. For health facility delivery as the outcome of interest, a cut-off point of less than 3 (or below) showed greater odds of institutional delivery, whereas those scoring above three were more likely to deliver at home (figure 3C-D). Similarly, in figure 4A-B, women in low vulnerability groups had a higher percentage of ANC visits, compared with higher vulnerability groups. In figure 4C-D, the composite score of the four items in urban–rural setting and six items in rural setting showed a steady decline in ANC visits with statistically significant values among women who have greater than one or more intersecting vulnerability score points (p<0.001). These findings indicate that the four and six items in urban–rural and rural settings, respectively, can segment women who are attending ANC less than the recommended number of visits. Odds of ANC visits with vulnerability score showed that the cut-off point of less than one indicated less vulnerability and a score of one or more indicated greater vulnerability (figure 4C-D).

**Figure 5:**
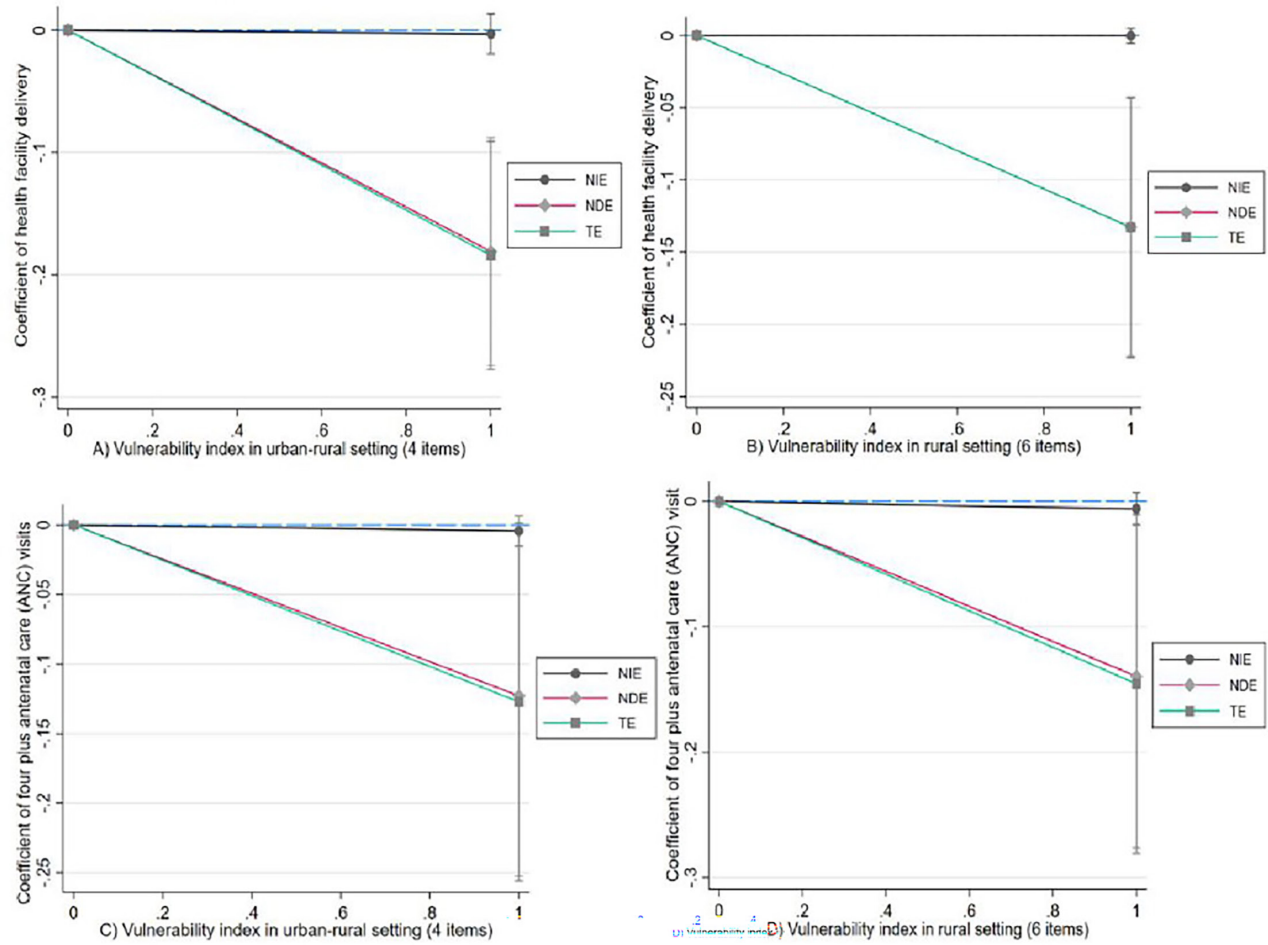
Mediation effect of ANC visit on the relationship between vulnerability scores and health facility delivery across urban–rural and rural settings. NDE, net direct effect; NIE, net indirect effect; TE, total effect.

A mediation regression analysis was done to depict the direct, indirect and total effects (TEs) of vulnerability indexes on maternal services, with ANC visit as a mediator. The effect of vulnerability on health facility delivery was mediated through ANC visits. As indicated in figure 5A and figure 5C (four items identified for urban–rural settings), women in higher vulnerability groups were less likely to make ANC visits and were less likely to deliver in health facilities, as compared with women in the low vulnerability groups (p<0.001 of net direct effect (NDE) and TE). However, the net indirect effect of vulnerability on health facility delivery mediating through effects of ANC was not significant (figure 5 of A). This implies that ANC may not be an effective pathway so that vulnerability scores influence health facility delivery. The vulnerability score has a direct significant effect on health facility delivery. Similarly, as shown in figure 5B and figure 5D (six items identified for rural setting), women living with vulnerability were less likely to go for ANC visits and less likely to deliver in health facilities, compared with women with low vulnerabilities (p<0.01 for both NDE and TE). This meditation could validate the odds of trend analysis (figure 3 C–D and figure 4 C-D).

## Discussion

The MVST, we introduced and tested in this paper, we believe, it is a practical and valuable tool for a number of reasons. First, literature indicates that interventions that are more narrowly targeted are likely to be more efficient and cost-effective than those that can only make a generic appeal.^34^,^36^ A tool like the MVST, which segments audiences based on their vulnerabilities, represents a crucial first step in designing tailored interventions. Identifying the segment to which a given pregnant woman belongs enables the development of targeted materials and strategies that resonate with her specific needs.^2037^,^39^

Second, conceptually (though not yet practically), this tool may be able to reduce disparities in maternal mortality. In Ethiopia, like in many other low- and middle-income countries, we have seen significant progress in reducing maternal mortality,^1 40^ but the prevailing maternal mortality rates are still much higher than developed countries.^41^,^43^ This is because of what we might call the *neglect at the margins*—while interventions, educational programmes and government policies have reduced the overall mortality, women at the edges of society, those who are subject to many intersecting vulnerability factors, have not been well served by existing policies and interventions. This is likely because of the unique constellation of vulnerability-enhancing factors that they face. Evidence shows that women experience various risks and barriers during pregnancy, resulting in adverse maternal and neonatal outcomes, such as pre-eclampsia, preterm birth and stillbirth.^44^ The MVST is designed to identify women at the highest levels of risk. While the tool, of course, does not develop specific interventions for them, it provides the first step in doing so—by identifying who the women are in a community that needs support.

Third, the MVST is meant to be an easy-to-use device that does not come with a large literacy burden. We envision that this tool will be used by frontline workers (in Ethiopia, they are often not highly educated) with relative ease to identify women who fall in the high-vulnerability category. Once they are identified, frontline workers can then be trained in addressing the unique challenges that the women face. Furthermore, this tool can be used in both an urban and a rural setting, places where some of the underlying challenges, vulnerability sources and deficits can be different.

We should note, however, that the MVST is for identifying women living with vulnerabilities; it is not an intervention strategy. Put another way, it is a population segmentation tool, not a tailoring tool. Hence, the next step for us is to test and scale the efficacy of this tool in a real world setting to learn whether it can effectively identify different population segments, and once so segmented, whether interventions can be designed to cater specifically to the different audience segments.

It is also worth pointing out that the MVST did not—deliberately so—take into account cultural practices and norms that could also be used to segment audiences. Our rationale for this decision was that we wanted to design a tool that could be used by frontline workers and other providers without having to collect extensive data; rather, by making use of available information, the tool was meant to provide meaningful audience profiles.

We recommend that the MVST be adopted by interventions in various regions of Ethiopia to address a number of research questions. For example, it would be useful to know whether, in fact, adopting the tool is more cost-effective than adopting the extant generic approach. While the tool can identify women with specific needs (eg, living at great distances from a health facility, not having the economic or financial means to arrange for transportation to the health facility for delivery), using the tool may itself require extra resources (eg, in terms of training of frontline workers) and it would be fruitful to assess whether the extra cost would result in better outcomes. Toward this end, it would also be useful to know whether frontline workers would see the utility of such a tool and whether they would be amenable to using it. Finally, future studies could ask and formally test the research question that would emerge from a primary assumption guiding this study: that a well-targeted intervention will be more effective (and perhaps even more cost-effective) than a generic (ie, a non-targeted) one.

The Ethiopian policy goal of achieving complete ANC attendance and institutional delivery in the country, though a noble one, is yet distal in attainment. Despite significant improvements in maternal health service coverage, disparities in MMRs have existed in the last decade.^1^ Existing gaps across both regions and socioeconomic conditions can only be reduced if interventions are tailored according to the specific individual, cultural, social and environmental circumstances of the target audience. By knowing more about the specific target audience, a process that can be facilitated by the MVST, it is our hope that more specifically tailored intervention can be developed and implemented. Additionally, Ethiopia has a community health extension programme that has been implemented through HEWs and community volunteer health workers (health development army) whose main functions are to conduct health promotion activities in a house-to-house visit or at health post level. Having a simple MVST device will enable these workers to easily identify women at higher vulnerability risk and connect them with the services. Moreover, our MVST device (automated digital tool) allows the frontline workers to easily segment pregnant women during their house-to-house visits, and it yields real-time segmentation unlike other population segmentation tools that require prior survey data collection and sophisticated segmentation analysis.^39 45 46^ The validity of this tool, however, will need further testing, and we hope other researchers will test its utility in other contexts, countries and settings.

We acknowledge certain limitations of our study. Given our focus on identifying those most disadvantaged in accessing care, our vulnerability criteria may not be universally applicable across broader regions of Ethiopia, where societal, cultural, economic and environmental vulnerabilities differ from our study sites. Nevertheless, we believe the process of developing a tool to identify the most invisible populations and refining it based on residential areas to enhance its practical utility can serve as a replicable approach in other contexts. Further, we acknowledge that maternal vulnerability is not a static concept. Rather, women’s levels and facets of vulnerability continuously evolve throughout the stages of pregnancy, childbirth and the postpartum period, all of which are influenced by pre-existing health conditions and their responses to emerging threats and barriers. Future research could benefit from refining our tool to capture vulnerability-enhancing factors unique to the different stages of pregnancy. Lastly, our study focused on maternal care-seeking behaviours as outcomes, emphasising its potential to address gaps in service coverage. While we consider service utilisation a strong predictor of maternal and child outcomes, we recommend testing the MVST against other health outcomes to uncover additional important vulnerability factors.

## Conclusion

To our knowledge, this is the first study that consolidates holistic factors affecting maternal health service utilisation to develop a tool that identifies the most underserved pregnant women in Ethiopia. While other vulnerability tools are extant, we believe that the MVST offers a targeted perspective by taking account of the health system’s contexts along with the sociocultural dimensions relevant to our target population. The MVST also ensures its practicality in the field, as it presents only the essential factors that define women’s vulnerability and fine-tunes its application based on residential areas. This approach results in a tool that can be used to inform effective, cost-saving intervention strategies, while minimising the burden on frontline workers and pregnant women during roll-out. It would be worth further developing and validating vulnerability segmentation tools for maternal and child health programming.

## Supplementary material

10.1136/bmjgh-2024-018811 online supplemental figure 1

## Data Availability

Data are available upon reasonable request.
