# Supplementary figures and images for: Introducing and testing the maternal vulnerability segmentation tool (MVST) for essential health services use: demonstrating its use in Oromia, Ethiopia
Source: BMJ Glob Health. 2026 Jan 7;11(1):e018811. doi: 10.1136/bmjgh-2024-018811 (PMC12781986; doi:10.1136/bmjgh-2024-018811)

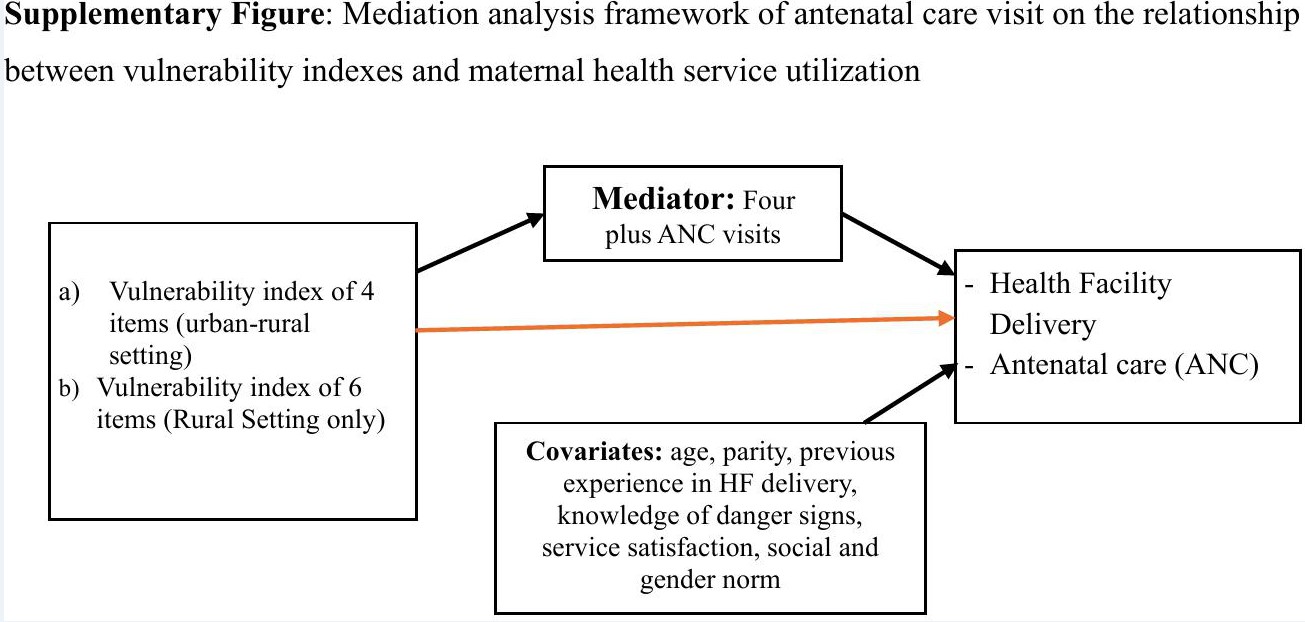

Supplement: online supplemental figure 1 [file bmjgh-11-1-s001.jpg]
